# Impact of early initiation of renin-angiotensin blockade on renal function and clinical outcomes in patients with hypertensive emergency: a retrospective cohort study

**DOI:** 10.1186/s12882-023-03117-1

**Published:** 2023-03-22

**Authors:** Keita Endo, Koichi Hayashi, Yuki Hara, Akihiro Miyake, Keisuke Takano, Takehiro Horikawa, Kaede Yoshino, Masahiro Sakai, Koichi Kitamura, Shinsuke Ito, Naohiko Imai, Shigeki Fujitani, Toshihiko Suzuki

**Affiliations:** 1 Department of Nephrology, Diabetes and Endocrinology, Tokyo Bay Urayasu Ichikawa Medical Center, Urayasu, Chiba Japan; 2 Department of Emergency and Critical Care Medicine, St Marianna University School of Medicine, 2-16-1 Sugao, Miyamae-Ku, Kawasaki, Kanagawa 216-8511 Japan; 3 Division of Nephrology and Hypertension, Department of Internal Medicine, St Marianna University School of Medicine, Kawasaki, Kanagawa Japan

**Keywords:** Antihypertensive drugs, Hypertensive emergencies, Kidney, Renal survival, Renin-angiotensin system, eGFR

## Abstract

**Background:**

Hypertensive emergency is a critical disease that causes multifaceted sequelae, including end-stage kidney disease and cardiovascular disease. Although the renin–angiotensin–aldosterone (RAA) system is enormously activated in this disease, there are few reports that attempt to characterize the effect of early use of RAA inhibitors (RASi) on the temporal course of kidney function.

**Methods:**

This retrospective cohort study was conducted to clarify whether the early use of RASi during hospitalization offered more favorable benefits on short-term renal function and long-term renal outcomes in patients with hypertensive emergencies. We enrolled a total of 49 patients who visited our medical center with acute severe hypertension and multiple organ dysfunction between April 2012 and August 2020. Upon admission, the patients were treated with intravenous followed by oral antihypertensive drugs, including RASi and Ca channel blockers (CCB). Kidney function as well as other laboratory and clinical parameters were compared between RASi-treated and CCB- treated group over 2 years.

**Results:**

Antihypertensive treatment effectively reduced blood pressure from 222 ± 28/142 ± 21 to 141 ± 18/87 ± 14 mmHg at 2 weeks and eGFR was gradually restored from 33.2 ± 23.3 to 40.4 ± 22.5 mL/min/1.73m^2^ at 1 year. The renal effect of antihypertensive drugs was particularly conspicuous when RASi was started in combination with other conventional antihypertensive drugs at the early period of hospitalization (2nd day [IQR: 1–5.5]) and even in patients with moderately to severely diminished eGFR (< 30 mL/min/1.73 m^2^) on admission. In contrast, CCB modestly restored eGFR during the observation period. Furthermore, renal survival probabilities were progressively deteriorated in patients who had manifested reduced eGFR (< 15 mL/min/1.73 m^2^) or massive proteinuria (urine protein/creatinine ≥ 3.5 g/gCr) on admission. Early use of RASi was associated with a favorable 2-year renal survival probability (0.90 [95%CI: 0.77–1.0] vs. 0.63 [95%CI: 0.34–0.92] for RASi ( +) and RASi (-), respectively, *p* = 0.036) whereas no apparent difference in renal survival was noted for CCB.

**Conclusions:**

Early use of RASi contributes to the renal functional recovery from acute reduction in eGFR among patients with hypertensive emergencies. Furthermore, RASi offers more favorable effect on 2-year renal survival, compared with CCB.

**Supplementary Information:**

The online version contains supplementary material available at 10.1186/s12882-023-03117-1.

## Background

Hypertensive emergency is an acute progressive disorder caused by severe hypertension and requires prompt recognition and timely intervention which could prevent the occurrence of catastrophic events, including cerebral hemorrhage, heart failure, and acute kidney impairment. This disorder is arbitrarily defined in many guidelines as acutely elevated blood pressure (BP), mostly more than 180/120 mmHg [1–3], with barotrauma on major vital organs [4]. Alternatively, malignant hypertension is characterized by acute severe hypertension with advanced retinopathy and has long been recognized as a subset of hypertensive emergency or crisis [1–3], which hence raises the possibility for need of reappraisal of malignant hypertension. Indeed, a growing number of studies have attempted to characterize the pathophysiology of the hypertensive emergency and have reported that hospital admissions for this disease are increasing during the past 20 years [5, 6]. Nevertheless, there have been a small number of investigations that evaluate the long-term effects of this acute event on target organs, particularly the kidney which may progress to end stage kidney disease (ESKD) requiring renal replacement therapy.

Great progress has been made regarding the development of antihypertensive drugs, and various drugs with different mechanisms are available and have been in clinical use solely or in combination with other types of antihypertensive agents. In malignant hypertension, long-term control of BP is reported to offer favorable renal outcomes [7]. Because the renin–angiotensin–aldosterone (RAA) system is activated at least in the early phase of this circumstance, several lines of studies suggest that RAA inhibitors (RASi’s) are a cornerstone of treating malignant hypertension [8, 9]. In contrast, it was also shown that no differences in efficacy nor safety were noted among antihypertensive drugs [10]. As a target organ for malignant hypertension or hypertensive emergencies, the kidney is of particular importance because it may progress to nephrosclerosis unless BP is well controlled [7]. Furthermore, since hypertensive emergencies usually cause accelerated kidney dysfunction, some concern arises that the treatment with RASi might further aggravate renal function [11] and elevate the risk for the progression toward chronic kidney disease (CKD) [12]. Nevertheless, there has been little evidence as to the association between the classes of antihypertensive drugs and their short/long-term effects on target organs, particularly the kidney, in patients with hypertensive emergencies.

In the present study, we conducted a 24-month follow-up evaluation of renal function as well as renal survival to clarify whether the early use of RASi during hospitalization effectively ameliorated the short- and long-term renal outcomes in patients with hypertensive emergencies.

## Methods

This study is a retrospective cohort analysis to investigate the effect of RASi, compared to calcium channel blockers (CCB’s), on the temporal course of renal function and 2-year renal outcomes in patients with hypertensive emergencies. The study was approved by the Ethics Committee of Tokyo Bay Urayasu-Ichikawa Medical Center with waiver of the requirement for obtaining informed consent (approval No. 726) and was registered at UMIN (ID#; UMIN000047340). The study was conducted in accordance with the Declaration of Helsinki. Information from medical records was anonymized and deidentified prior to final analysis.

### Study population

During the period between April 2012 and August 2020, the patients who visited the emergency department of our hospital with severe hypertension (systolic BP ≥ 180 mmHg or diastolic BP ≥ 120 mmHg) were screened for possible possession of hypertension-induced organ damage, including renal impairment, heart failure, stroke, retinopathy and thrombotic microangiopathy. A total of 77 patients met the inclusion criteria based on various clinical and laboratory examination. Among them, 28 patients were excluded; 5 patients died upon the hospitalization, 18 patients were transferred to other hospitals from our emergency room without admission, and 5 patients required maintenance dialysis therapy upon hospitalization. Ultimately, 49 patients were deemed eligible for further analysis.

### Study design

Upon admission, the patients were treated with intravenous followed by oral antihypertensive drugs [1], and the types and doses of antihypertensive drugs were modified based on the level of BP during hospitalization. BP and laboratory data, including serum creatinine, hematocrit and proteinuria, were serially evaluated over 24 months. Hormonal parameters, including plasma renin activity and aldosterone (Ald), cardiac ultrasound and the eye ground were examined on admission. The incidence of moderately to severely impaired renal function (estimated glomerular filtration rate (eGFR) less than 30 mL/min/1.73 m^2^) and cardiovascular complications such as heart failure, myocardial infarction, cerebral infarction, and thrombotic microangiopathy (TMA) was also evaluated. TMA was determined by the presence of microangiopathic hemolytic anemia and thrombocytopenia (platelet count less than 15 × 10^4^/mm^3^) [13]. Hypertensive retinopathy was evaluated with Scheie’s classification. Plasma Ald was measured with a traditional RIA method.

The antihypertensive drugs used at the time of discharge from hospital were identified as RASi (ACE inhibitors and ARB) or CCB. The patients who had received or started RASi by the time of discharge were referred to as RASi( +) group while those who were not given RASi at the time of discharge were defined as RASi(-) group. The effects of the antihypertensive agents on BP, eGFR and urinary protein/creatinine ratio (urine-P/Cr) were serially assessed over 24 months. eGFR was calculated using the formula adapted to the Japanese population [14]. eGFR = 194 × age^−0.287^ × serum creatinine.^−1.094^ (× 0.739 if female)

### Statistical analysis

The results are expressed as the mean ± standard deviation (SD), or the median [lower quartile-upper quartile]. Data were compared with the Student’s t-test or the Mann–Whitney U test. Serial changes in eGFR over 2 years were assessed with linear regression analysis. Multiple regression analyses were applied to evaluate independent predictors for the changes in eGFR. The serial (i.e., 1, 3, 6, 12 and 24 months from admission) changes in the regression coefficients were evaluated as a function of the following independent parameters; systolic BP, initial eGFR, Ald levels on admission, and antihypertensive drugs used. The chi-square or Fisher’s exact test was used to compare categorical variables, including the number of patients. Kaplan–Meier analysis was used to generate renal survival curves. Comparison between two survival curves was made using the log-rank test. Statistical analyses were performed using IBM SPSS Statistics (version 25). P values less than 0.05 were considered statistically significant.

## Results

### Patient characteristics

The prevalence of hypertensive emergencies showed that males predominated (75.5%) and BP was markedly elevated, exceeding 200/130 mmHg (Table 1). Although 79.6% of the patients were aware of having hypertension, only 17.9% of these subjects actually received antihypertensive treatment. In the RASi ( +) group, 3 patients had been receiving RASi at the time of admission. There was no difference in the incidence of diabetes or smoking habit between RASi ( +) and RAS(-) group. Although the medical information was obtained from a limited number of patients, the available data from previous medical check-up indicated that most of the patients were assumed to have essential hypertension but no CKD. Primary aldosteronism or renal vascular stenosis was not included based on clinical examination.

**Table 1:** Baseline patient profiles

	**Total (*n* = 49)**	**RASi(+) (*n* = 37)**	**RASi(-) (*n* = 12)**	***p* value**
Age (y/o)	47.2 ± 11.2	46.6 ± 11.8	49.3 ± 9.5	0.435
Male/female, n (male%)	37/12 (75.5%)	28/9 (75.7%)	9/3 (75.0%)	1.0
Systolic BP (mmHg)	222 ± 28	220 ± 26	231 ± 32	0.364
Diastolic BP (mmHg)	142 ± 21	144 ± 22	139 ± 18	0.323
Heart rate (beats/min)	106 ± 19	109 ± 19	99 ± 17	0.081
BMI (kg/m^2^)	29.5 ± 6.9	29.9 ± 7.4	28.4 ± 5.3	0.675
Previous hypertensive therapy, n (%)				
Aware of hypertension	39 (79.6%)	29 (78.4%)	10 (83.3%)	1.0
Duration of hypertension	7.6 ± 9.3 (y)	5.7 ± 6.4 (y)	13.2 ± 13.8 (y)	0.127
No treatment	32 [82.1%]	25 [86.2%]	7 [70.0%]	0.344
Under treatment	7 [17.9%]	4 [13.8%]	3 [30.0%]	
with RASi	5	3	2	1.0
CCBs	6	3	3	1.0
β blockers	1	0	1	0.429
Diuretics	2	1	1	1.0
Diabetes, n (%)	4 (8.2%)	3 (8.1%)	1 (8.3%)	1.0
Smoking, n (%)	26 (53.1%)	21 (56.8%)	5 (41.7%)	0.508

*BP* blood pressure, *BMI* body mass index, *RASi* renin-angiotensin system inhibitors, *CCBs* Ca channel blockers

On admission, mild anemia was seen and LDH was slightly elevated, but platelet count remained within the normal range (Table 2). eGFR was moderately to severely reduced and a subnephrotic range of proteinuria was observed. Both plasma renin activity and plasma Ald concentrations were markedly elevated. None of the parameters on admission was significantly different between RASi ( +) and RASi (-) group.

**Table 2:** Laboratory parameters on admission

	**Total (*n* = 49)**	**RASi(+) (*n* = 37)**	**RAS(-) (*n* = 12)**	***p* value**
Hematocrit (%)	37.9 ± 7.8	37.8 ± 8.3	37.6 ± 5.9	0.930
Platelet count (× 10^4^/mm^3^)	20.4 ± 7.9	20.0 ± 8.4	21.8 ± 6.3	0.494
LDH (U/L)	447 ± 354	493 ± 391	307 ± 132	0.114
Total protein (g/dL)	6.7 ± 0.9	6.7 ± 0.2	6.5 ± 0.3	0.484
Serum potassium (mEq/L)	3.8 ± 0.9	3.7 ± 0.9	4.1 ± 0.8	0.176
LDL-cholesterol (mg/dL)	121 ± 29	120 ± 26	123 ± 40	0.814
HDL-cholesterol (mg/dL)	45 ± 12	46 ± 12	42 ± 13	0.393
Hemoglobin A1c (%)	5.4 ± 1.0	5.4 ± 1.1	5.6 ± 0.6	0.489
eGFR (mL/min/1.73m^2^)	33.2 ± 23.3	35.1 ± 22.6	27.3 ± 25.5	0.319
Urine protein (g/gCr)	3.11 ± 3.29	2.61 ± 2.76	3.23 ± 2.61	0.148
Plasma renin activity (ng/mL/hr)	26 ± 38	30 ± 43	12 ± 12	0.134
Plasma aldosterone (pg/mL)	290 ± 243	329 ± 265	173 ± 100	0.055

*LDH* lactate dehydrogenase

### Adverse events

Renal impairment with eGFR less than 30 mL/min/1.73 m^2^ was the most frequently observed complication at the time of hospital admission (i.e., 51.0%), and ESKD ensued in 6 patients during the 2-year observational period (Table 3). Heart failure with reduced ejection fraction (< 40%) was observed in 8 patients (i.e., 16.3%).

**Table 3:** Major adverse events

Adverse events		n (%)
**Kidney**	Impaired renal function (eGFR < 30 mL/min/1.73 m^2^)	25 (51.0%)
	ESKD (dialysis)	6 (12.2%)
**Heart**	Heart failure (Ejection fraction < 40%)	8 (16.3%)
	NSTEMI	2 (4.1%)
**Brain**	Hemorrhage	0 (0%)
	Infarction	2 (4.1%)
	Subarachnoid hemorrhage	0 (0%)
	Encephalopathy	1 (2.0%)
**Vessels**	TMA	5 (10.2%)
**Eye ground**	H0/H1/H2/H3/H4	3/ 3/ 2/ 22/ 8
	Not evaluated	11

*NSTEMI* non-ST-segment elevation myocardial infarction, *AKI* acute kidney injury, *ESKD* end-stage kidney disease, *TMA* thrombotic microangiopathy

TMA developed in 5 cases (Table 3), who had higher plasma Ald levels (321 [IQR: 298–591] pg/mL) than the remaining population (201 [IQR: 131–322] pg/mL, *p* = 0.017). Among 38 patients who received fundoscopic evaluation on admission, 30 subjects (i.e., 78.9%) had severer retinal damage (≥ H3) and manifested higher Ald levels (301 [IQR: 173–450] pg/mL) than those with less retinal damage (136 [IQR: 123–164] pg/mL, *p* = 0.006).

### Serial changes in BP and other parameters

Both systolic and diastolic BP were strikingly decreased within 2 weeks of hospitalization (141 ± 18/87 ± 14 mmHg, *p* < 0.001) and were maintained constant thereafter (Table 4). Hematocrit tended to decrease at 2 weeks and then gradually elevated, the changes of which, however, did not attain statistical significance. The eGFR was gradually elevated with a significant increase observed at 2 months (37.9 ± 23.3 mL/min/1.73 m^2^, *p* = 0.016 vs. 0 month), and remained elevated throughout the study period. Urine-P/Cr was decreased, which paralleled the changes in BP. Kaplan–Meier analysis showed a modest reduction in renal survival probability during the 24-month observational period (0.82 [95%CI: 0.68–0.95]).

**Table 4:** The changes in blood pressure and renal parameters over 24 months

	**Months**							
**Parameters**	0	0.5	1	2	3	6	12	24
**Systolic BP** (mmHg)	222 ± 28	141 ± 18*	141 ± 18*	138 ± 16*	138 ± 22*	137 ± 24*	140 ± 20*	135 ± 14*
**Diastolic BP** (mmHg)	142 ± 21	87 ± 14*	84 ± 13*	82 ± 11*	83 ± 14*	83 ± 18*	84 ± 11*	75 ± 17*
**Hematocrit** (%)	37.9 ± 7.8	35.6 ± 7.1	35.9 ± 6.3	36.0 ± 6.0	36.7 ± 5.7	38.1 ± 5.0	38.8 ± 5.0	40.0 ± 5.8
**eGFR** (mL/min/1.73 m^2^)	33.2 ± 23.3	33.7 ± 23.5	36.0 ± 23.2	37.9 ± 23.3^#1^	37.8 ± 20.3	40.8 ± 21.8^#2^	40.4 ± 22.5^#3^	46.5 ± 21.7^#4^
**Urine-P/Cr** (g/gCr)	3.11 ± 3.29	1.43 ± 1.28*	1.23 ± 1.60*	1.14 ± 1.31*	0.92 ± 1.08*	0.901.36*	0.60 ± 0.75*	0.60 ± 0.76*
**Renal survival probability** [95% CI]	1.0	1.0	1.0	0.98 [0.93–1.0]	0.98 [0.93–1.0]	0.98 [0.93–1.0]	0.90 [0.8–0.99]	0.82 [0.68–0.95]

*BP* blood pressure, *urine-P/Cr* urinary protein/creatinine ratio

^*^; *p* < 0.001 vs 0 month

^#^1; *p* = 0.016

^#^2; *p* = 0.003

^#^3; *p* = 0.019

^#^4; *p* = 0.018 vs 0 month

### Effects of antihypertensive drugs on serial changes in eGFR

At the time of hospital discharge, the patients received 3 [IQR: 2.0–3.0] different types of antihypertensive drugs (RASi; *n* = 37, CCB; *n* = 42, β blockers; *n* = 20, α blockers; *n* = 6, diuretics; *n* = 17). Because RASi and CCB strikingly affected renal hemodynamic function, the impact of these drugs on eGFR was serially assessed over 2 years.

**Fig. 1:**
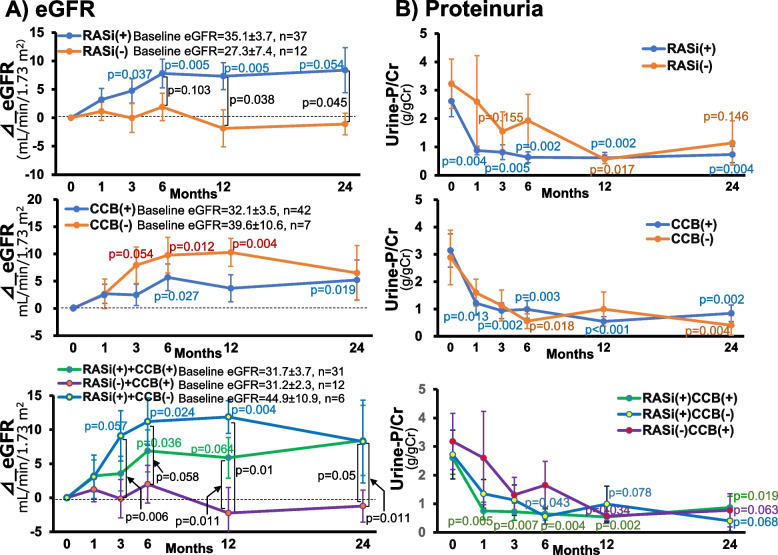
Effects of antihypertensive drugs on changes in eGFR and proteinuria over 24 months RASi; renin-angiotensin system inhibitor, CCB; Ca channel blocker, Δ_eGFR; changes in eGFR from 0 month, urine-P/Cr; urinary protein/creatinine ratio

Figure 1 illustrates the changes in eGFR and urine-P/Cr in patients receiving RASi and/or CCB during the observational period; RASi was initiated early during hospitalization (i.e., 2^nd^ day [IQR: 1.0–5.5] of the admission). Obviously, the RASi therapy progressively elevated eGFR during the first 6 months of admission and then maintained eGFR constant until the end of the observational period (Fig. 1A); no cases showed a rapid decline in eGFR that necessitated the discontinuation of RASi. In contrast, CCB (amlodipine; *n* = 28, nifedipine CR; *n* = 14) did not modify the temporal course of eGFR over 24 months. Finally, the interactive effects of RASi and CCB indicated that the RASi therapy, whether or not in combination with CCB, elicited a recovery of eGFR from the kidney impairment induced by hypertensive emergencies. Similarly, early decreases in urine-P/Cr was observed in patients treated with RASi, which did not depend on whether CCB was co-treated or not (Fig. 1B). No difference in BP was observed between RASi( +) and RASi(-) group, between CCB( +) and CCB(-) group, or among the three groups (i.e., RASi, CCB, RASi + CCB, Supplementary Fig. S1).

We also conducted multiple regression analysis at each time point to clarify which parameters contributed to the changes in eGFR from admission. Thus, among the antihypertensive drugs, RASi was the only variable that significantly affected the changes in eGFR at 1 month and the coefficient for RASi tended to increase with time whereas that for CCB or other antihypertensive agents had no appreciable effect on the change in eGFR (Supplementary Table S1).

### Changes in eGFR and hematocrit in patients with moderately to severely reduced eGFR on admission

**Fig. 2:**
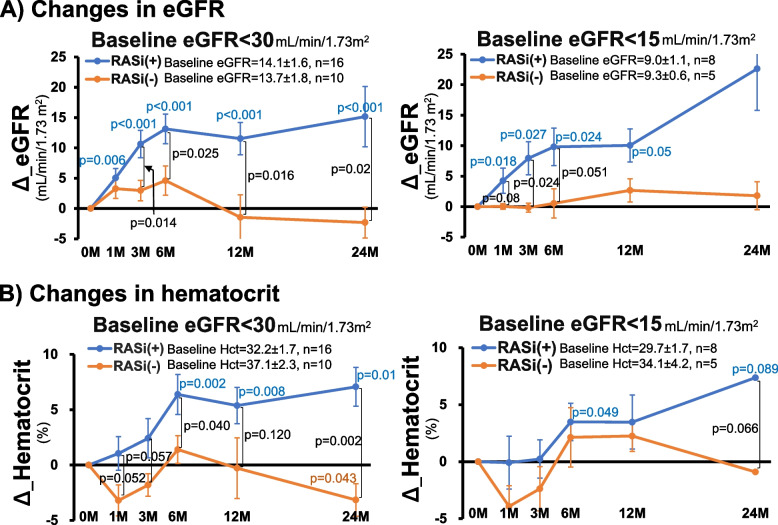
Effects of RASi on the changes in eGFR and hematocrit in patients with reduced eGFR. RASi; renin-angiotensin system inhibitor, Δ_eGFR; changes in eGFR from 0 month, Hct; hematocrit

The effect of RASi on the changes in eGFR was evaluated in patients with eGFR < 30 mL/min/1.73 m^2^ on admission. After 3 months of RASi therapy, a greater rise in eGFR was observed, compared with that seen in patients without RASi treatment (*p* = 0.014), and this difference was maintained throughout the subsequent period (Fig. 2A). A similar trend was seen when evaluated in patients with more advanced renal impairment (eGFR < 15 mL/min/1.73 m^2^). The changes in hematocrit nearly paralleled those in eGFR (Fig. 2B).

### Factors affecting 2-year renal survival

The impact of various parameters on 2-year renal survival rates was assessed. Both severely diminished eGFR (< 15 mL/min/1.73 m^2^) and greater urine-P/Cr (≥ 3.5 g/gCr) seen on admission were associated with reduced renal survival probabilities whereas plasma Ald had no effect on the subsequent renal survival (Supplementary Fig. S2). Higher follow-up systolic BP (≥ 135 mmHg) tended to reduce renal survival probability, which however did not reach statistical significance.

**Fig. 3:**
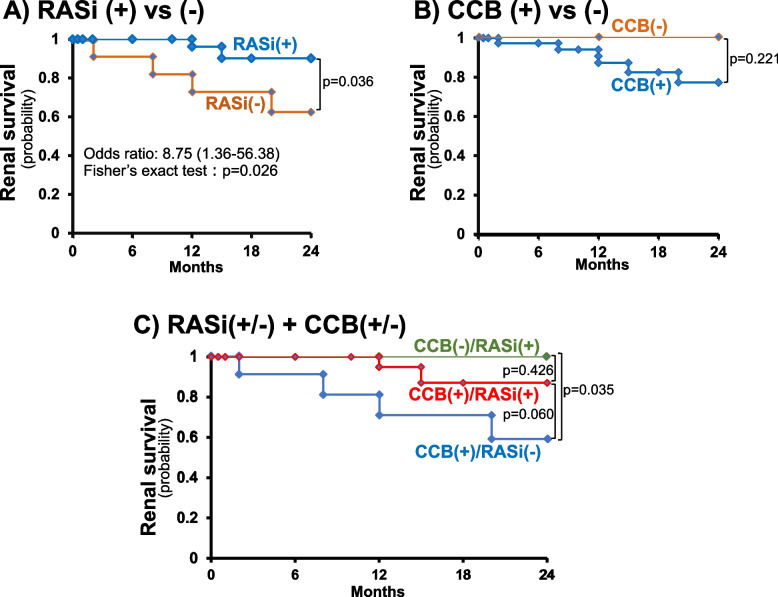
Association between renal survival and various antihypertensive drugs during follow-up. RASi; renin-angiotensin system inhibitor, CCB; Ca channel blocker

When evaluated based on the antihypertensive drugs used, the renal survival probability was well preserved in patients who received RASi (0.90 [95%CI: 0.77–1.0] vs. 0.63 [95%CI: 0.34–0.92], *p* = 0.036), with an odds ratio for renal survival of 8.75 [95%CI: 1.36–56.38] (Fig. 3A). In contrast, CCB failed to affect the renal survival probabilities (Fig. 3B). Finally, among the CCB-treated groups, the simultaneous treatment with RASi tended to improve the 2-year renal survival (0.87 [95%CI: 0.70–1.0] vs. 0.59 [95%CI: 0.28–0.90], for CCB( +)/RASi( +) and CCB( +)/RASi(-), respectively, *p* = 0.060, Fig. 3C). Among the patients treated with RASi, in contrast, the co-treatment with CCB did not affect the renal survival probabilities (CCB( +)/RASi( +) vs. CCB(-)/RASi( +), *p* = 0.426).

### Effect of the timing of RASi administration on the recovery of renal function

**Fig. 4:**
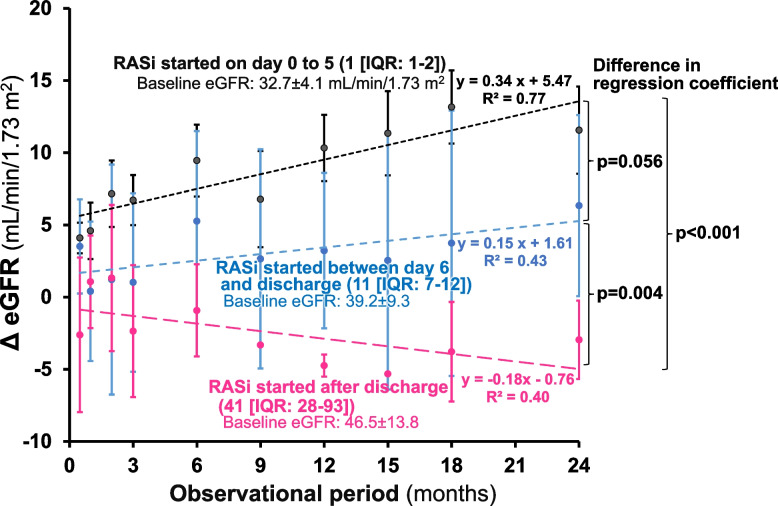
Impact of the timing of RASi initiation on the changes in eGFR. RASi; renin-angiotensin system inhibitor, Δ_eGFR; changes in eGFR from 0 month

We further evaluated the effect of RASi on the changes in eGFR, based on the timing of the RASi initiation, i.e., starting RASi on 0 to 5 hospital day (day 1 [IQR: 1–2], *n* = 25; very early) or on day 6 or later during hospitalization (day 11 [7–12], *n* = 9, early). Furthermore, among the RASi(-) group (*n* = 12), 5 patients actually started RASi after hospital discharge (day 41 [28-93]; late). The BP was reduced in a similar manner among the 3 groups (data not shown). In contrast, Thus, there was observed a greater upward tendency of the change in eGFR in patients receiving RASi very early (regression coefficient = 0.34), compared with those who were given RASi early (regression coefficient = 0.15, *p* = 0.056) or late (regression coefficient = -0.18, *p* < 0.001, Fig. 4).

## Discussion

Since the introduction of malignant hypertension [15], abundant evidence has been accumulated showing the devastating prognosis involving the damage of multiple target organs such as the kidney, the heart, the eye and the vasculature. Recently, the term’hypertensive emergency’ has been used more frequently, which consists of acute severe hypertension with multiple organ damage and hence encompasses the disease entity of malignant hypertension. With this definition, we attempted to characterize the clinical features underlying the acute hypertensive organ damage, including the renal injury. Furthermore, the impact of antihypertensive drugs, particularly RASi, on the recovery process from acute hypertensive renal injury and the subsequent renal outcomes was assessed.

### Hypertensive emergencies and organ damage

The present study has demonstrated a marked elevation in BP and multiple target organ damage, including the renal injury, on admission (Tables 1–3). Whereas BP was controlled rapidly, renal function improved gradually, which lasted for more than one year (Table 4). Furthermore, the renal survival was well preserved throughout the 24-month study period. Hence, the recovery from the acute kidney damage suggests that the renal injury is mediated in part by reversible or modifiable factors, including severe ischemia [16], transient endothelial injury [6, 17] and hyperperfusion-induced capillary leakage [17].

Regarding the major adverse events induced by hypertensive emergencies, impaired renal function and heart failure were observed more frequently (Table 3). Furthermore, among 38 patients who underwent fundoscopic examination, 30 cases had severe damage (i.e., grade H3/H4). The impairment in these organs is most likely associated with severe hypertension, but elevated Ald may also be responsible for severe retinopathy (grade H3/H4: 301 [173–450] pg/mL) and TMA (321 [298–591] pg/mL). Since advanced retinopathy and TMA are attributed to vascular and/or endothelial damage, Ald may play an additive role in the development of the hypertensive injury [13, 18–20], which is consistent with the previous reports showing Ald-mediated hypertensive endothelial injury independent of renin or angiotensin [21, 22].

### Early RASi treatment and renal function

Although the progress in drug development facilitates efficient management of BP in hypertensive emergencies [4, 8], controversy remains as to whether the early blockade of the RAA system confers short- and long-term benefits on target organs [8–10]. The present study attempts to elucidate whether RASi, which is established to alleviate the progression of CKD, contributes to the recovery process of renal function following hypertensive emergencies. Thus, the treatment with RASi, when started early during hospitalization, elicited a progressive increase in eGFR during the first 6 months following the initiation of antihypertensive therapy (Fig. 1A). Even in patients with moderately to severely impaired renal function, the same tendency was noted along with similar changes in hematocrit (Fig. 2). In patients with no RASi treatment, by contrast, eGFR remained unaltered throughout the observational period. Hence, it follows that the treatment with RASi constitutes a critical determinant of the recovery from the renal injury induced by hypertensive emergencies. Furthermore, the early decrease in proteinuria observed with RASi (Fig. 1B) is not attributed to the glomerular hemodynamic action inherent in RASi (i.e., a reduction in glomerular pressure) but rather may reflect the recovery process from the renal injury caused by hypertensive emergencies. Finally, in patients with impaired renal function, the treatment with RASi is associated with an early increase in hematocrit (Fig. 2B). This may be attributed to ameliorated renal injury or depressed activity of TMA since the treatment of hypertensive emergencies results in reduced RAA activity and correction of hemoconcentration, both of which should lead to a decrease in hematocrit.

Although the RAA system is markedly activated in hypertensive emergencies, very few studies have reported early use of RASi because of a potential risk of a rapid BP decline [9, 23, 24]. Rubin et al. [9] demonstrated that the use of RASi at the acute phase with a very low dose was effective for controlling malignant hypertension. There is a case report showing that initiation of RASi on day 14 is associated with successful recovery of the renal function which persisted over 18 months [23]. In the present study, the use of RASi was commenced very early during hospitalization (2^nd^ day [IQR: 1.0–5.5]). When compared based on the day of RASi initiation (i.e., very early [day 0–5] vs. early [day 6 to discharge] or late [after hospital discharge]), the trend in the increases in eGFR over 24 months was greater when RASi was given very early after admission (Fig. 4). This finding however requires more solid confirmation.

Of note, 51.0% of the patients exhibited impaired renal function on admission (Table 3). There is a growing number of studies showing a beneficial role of RAA blockade in preventing the transition of AKI to CKD [25–27]. Chou et al. [25] demonstrated the use of RASi significantly reduced the rate of ensuing CKD in patients who had suffered cardiac surgery-associated AKI. Experimental studies also showed that RASi or genetical angiotensin II type 1a loss prevented chronic tubulointerstitial damage induced by ischemia reperfusion AKI [26, 27]. Although diverse mechanisms trigger off AKI, activated RAA is suggested to be responsible at least in part for the transition of AKI to CKD [28]. Hence, to the extent that both acute tubular injury and chronic tubulointerstitial damage coexist in malignant nephrosclerosis [29], more favorable effects of RASi might be anticipated when administered early during hospitalization (Figs. 1–3) [23]. Alternatively, RASi might protect against vascular injury induced by elevated aldosterone [21, 22]. Of note, there exists some controversy as to whether the use of RASi after AKI is associated with higher incidence of recurrent AKI and the subsequent risk for developing CKD [11, 12, 30]. Although the present study, as well as a case report by Watanabe et al. [23], did not show a sudden decline in eGFR requiring discontinuation of RASi, these important issues need to be clarified in future studies.

### Comparison of renal protective effects of RASi and other antihypertensive drugs

Among antihypertensive drugs, both RASi and CCB not only exert profound effects on BP but also alter renal microvascular tone [31, 32] that could affect long-term renal function [33, 34]. The present study showed that the treatment with CCB had no effect on the temporal changes in eGFR (Fig. 1). To eliminate the confounding effects of the interaction between RASi and CCB, we assessed the effect of each agent or the combination therapy on the changes in eGFR. Thus, the patients treated with RASi, whether administered solely or in combination with CCB, manifested a gradual rise in eGFR during the initial 6 months and exhibited sustained elevations thereafter whereas CCB per se was able to maintain eGFR but failed to increase eGFR. Furthermore, multiple regression analyses indicate the greatest impact of RASi on the rise in eGFR at 1 month among the antihypertensive drugs and its role is growing with time whereas no significant correlation is seen with CCB or other antihypertensive agents (Supplementary Table S1). Collectively, these findings lend support to the premise that RASi not only contributes to the recovery process from severe hypertension-induced renal damage but is also responsible for the long-term renal outcomes.

### Association of 2-year renal survival with clinical parameters or antihypertensive therapies

The hypertensive emergency causes acute renal impairment, which, in some cases, deteriorates into ESKD requiring renal replacement therapy [7, 24]. Our current study reveals that severely impaired renal function (eGFR < 15 mL/min/1.73 m^2^) and heavy proteinuria (urine-P/Cr ≥ 3.5 g/gCr) seen on admission are associated with the downward progression toward ESKD (Supplementary Fig. S2). Plasma Ald levels on admission, however, do not affect the long-term renal outcomes, possibly because of the length of the period during which the kidney is exposed to high Ald levels. In primary aldosteronism, highly sustained Ald levels are associated with the elevated risk for developing CKD whereas surgical adrenalectomy mitigates the risk for the progression toward CKD [35].

Regarding BP control, Amraoui et al. [7] reported that the BP lower than 140/90 mmHg during the follow-up of malignant hypertensive patients was associated with lower incidence of renal end point. In the present study, however, lowering BP (< 135 mmHg) shows only a slight tendency to confer a benefit on the renal survival (*p* = 0.194, Supplementary Fig. S2D), thus suggesting a need for more expanded observations.

Consistent with the changes in eGFR, the early treatment with RASi is associated with more favorable renal outcomes than that without RASi, irrespective of simultaneous use of CCB (Fig. 3). It is reasonably inferred therefore that the renal recovery, as represented by the early elevation in eGFR, and the long-term renal protective effect of RASi warrant the early initiation of RASi in the treatment of hypertensive emergencies.

### Limitations

The results of the present study are obtained at a single medical facility in the urban area. The patients’ characteristics may therefore affect the medication adherence as well as the chance for regular medical checkups, which might influence the incidence of hypertensive emergencies. Although regular medical checkup systems are widely distributed in Japan and, indeed, 79.6% of the patients were aware of having hypertension, most patients are reluctant to receive antihypertensive therapy (Table 1). Second, physicians tend to withhold the administration of RASi to patients with advanced and acute renal dysfunction. While controversy exists regarding the prescription of RASi in advanced CKD [36–39], the present study suggests a critical role of RAA blockade in the recovery from acute renal impairment induced by hypertensive emergencies.

## Conclusions

Despite tremendous progress in the management of BP, the incidence of the hypertensive emergency does not decrease. Early initiation of RASi may play an important role in the renal recovery and, consequently, the long-term renal prognosis in patients with hypertensive emergencies. Large-scale and more long-term studies are required to dissipate the concern that early initiation of RASi may increase the risk of further rapid renal impairment in patients with hypertensive emergencies.

## Supplementary Information

**Additional file 1: Supplementary Table S1.** Multiple regression analysis for evaluation of the impact of antihypertensive drugs on the changes in eGFR from baseline.

**Additional file 2: Supplementary Fig. S1.** Temporal changes in blood pressure over 24 months. SBP; systolic blood pressure, DBP; diastolic blood pressure, RASi; renin-angiotensin system inhibitor, CCB; Ca channel blocker.

**Additional file 3: Supplementary Fig. S2.** Renal survival probabilities during 24-month follow-up. Urine-P/Cr; urinary protein/creatinine ratio, Ald; aldosterone, SBP; systolic blood pressure.

## Data Availability

The datasets generated and/or analyzed during the current study are not publicly available due to limitations of ethical approval and anonymity constraints involving the patient data, but are available from corresponding author on reasonable request.

## Abbreviations

BP: Blood pressure
ESKD: End stage kidney disease
RAA: Renin–angiotensin–aldosterone
RASi: Renin-angiotensin system inhibitor
CKD: Chronic kidney disease
CCB: Ca channel blocker
TMA: Thrombotic microangiopathy
AKI: Acute kidney injury
