# Supplementary material for: Impact of early initiation of renin-angiotensin blockade on renal function and clinical outcomes in patients with hypertensive emergency: a retrospective cohort study
Source: BMC Nephrol. 2023 Mar 22;24:68. doi: 10.1186/s12882-023-03117-1 (PMC10035153; doi:10.1186/s12882-023-03117-1)
Supplement: Supplementary file 1 — Additional file 1: Supplementary Table S1. Multiple regression analysis for evaluation of the impact of antihypertensive drugs on the changes in eGFR from baseline. [file 12882_2023_3117_MOESM1_ESM.pptx]

## Slide 1
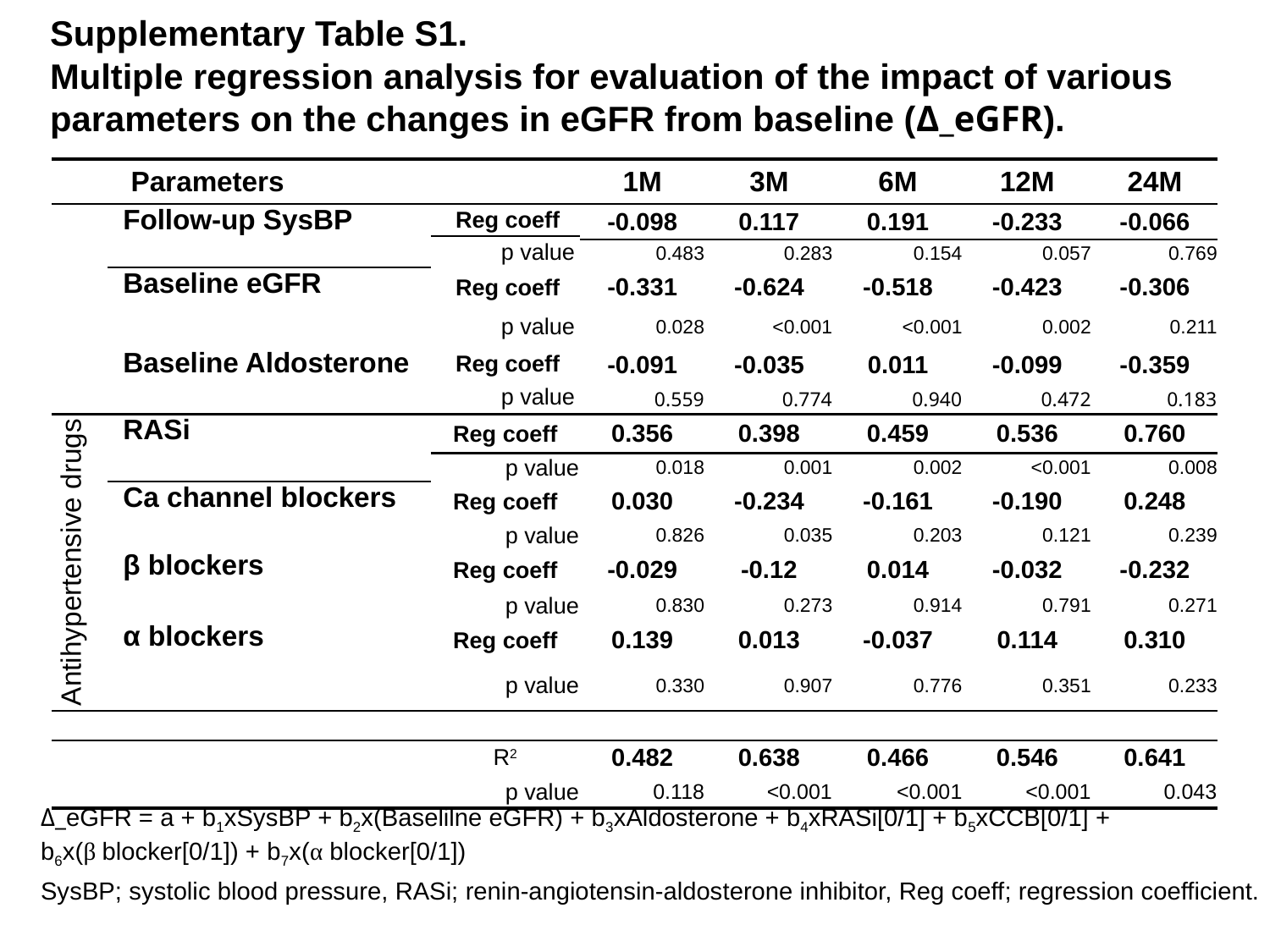

Supplementary Table S1.
Multiple regression analysis for evaluation of the impact of various parameters on the changes in eGFR from baseline (Δ_eGFR).
| Parameters | | | 1M | 3M | 6M | 12M | 24M |
| --- | --- | --- | --- | --- | --- | --- | --- |
| | Follow-up SysBP | Reg coeff | -0.098 | 0.117 | 0.191 | -0.233 | -0.066 |
| | | p value | | | | | |
| | | | 0.483 | 0.283 | 0.154 | 0.057 | 0.769 |
| Baseline | Baseline eGFR | Reg coeff | -0.331 | -0.624 | -0.518 | -0.423 | -0.306 |
| | | p value | 0.028 | <0.001 | <0.001 | 0.002 | 0.211 |
| | Baseline Aldosterone | Reg coeff | -0.091 | -0.035 | 0.011 | -0.099 | -0.359 |
| | | p value | | | | | |
| | | | 0.559 | 0.774 | 0.940 | 0.472 | 0.183 |
| Antihypertensive drugs | RASi | Reg coeff | 0.356 | 0.398 | 0.459 | 0.536 | 0.760 |
| | | p value | 0.018 | 0.001 | 0.002 | <0.001 | 0.008 |
| | Ca channel blockers | Reg coeff | 0.030 | -0.234 | -0.161 | -0.190 | 0.248 |
| | | p value | 0.826 | 0.035 | 0.203 | 0.121 | 0.239 |
| | β blockers | Reg coeff | -0.029 | -0.12 | 0.014 | -0.032 | -0.232 |
| | | p value | 0.830 | 0.273 | 0.914 | 0.791 | 0.271 |
| | α blockers | Reg coeff | 0.139 | 0.013 | -0.037 | 0.114 | 0.310 |
| | | p value | 0.330 | 0.907 | 0.776 | 0.351 | 0.233 |
| | | | | | | | |
| | | R2 | 0.482 | 0.638 | 0.466 | 0.546 | 0.641 |
| | | p value | 0.118 | <0.001 | <0.001 | <0.001 | 0.043 |
Δ_eGFR = a + b1xSysBP + b2x(Baselilne eGFR) + b3xAldosterone + b4xRASi[0/1] + b5xCCB[0/1] + b6x(β blocker[0/1]) + b7x(α blocker[0/1])
SysBP; systolic blood pressure, RASi; renin-angiotensin-aldosterone inhibitor, Reg coeff; regression coefficient.
