# Supplementary material for: Impact of early initiation of renin-angiotensin blockade on renal function and clinical outcomes in patients with hypertensive emergency: a retrospective cohort study
Source: BMC Nephrol. 2023 Mar 22;24:68. doi: 10.1186/s12882-023-03117-1 (PMC10035153; doi:10.1186/s12882-023-03117-1)
Supplement: Supplementary file 2 — Additional file 2: Supplementary Fig. S1. Temporal changes in blood pressure over 24 months. SBP; systolic blood pressure, DBP; diastolic blood pressure, RASi; renin-angiotensin system inhibitor, CCB; Ca channel blocker. [file 12882_2023_3117_MOESM2_ESM.pptx]

## Slide 1
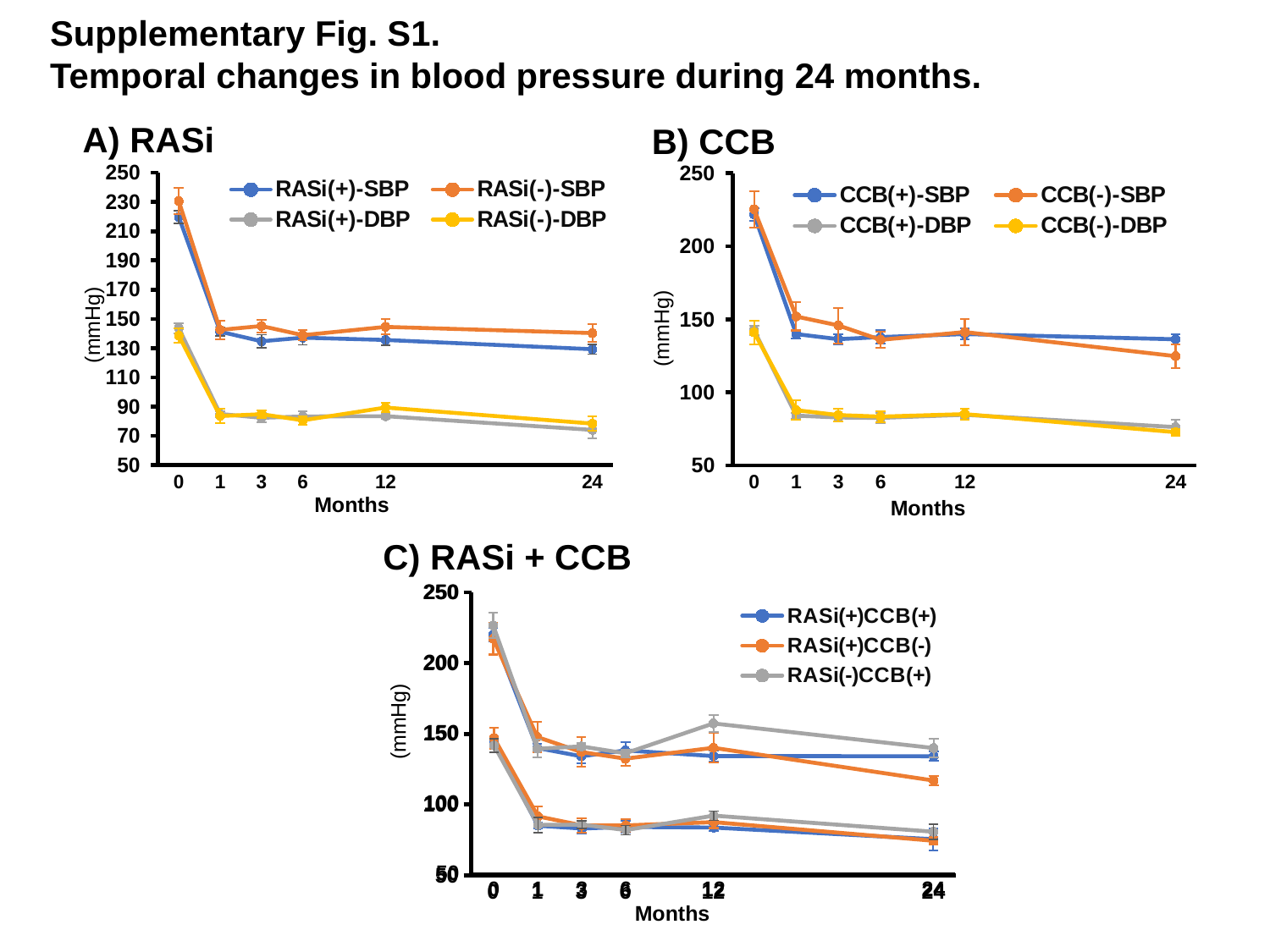

Supplementary Fig. S1.
Temporal changes in blood pressure during 24 months.
A) RASi
B) CCB
### Chart
| Category | | | | |
|---|---|---|---|---|
| 0 | 219.72972972972974 | 230.58333333333334 | 143.64864864864865 | 138.5 |
| 1 | 141.28125 | 142.54545454545453 | 85.0 | 83.54545454545455 |
| 3 | 134.71428571428572 | 145.16666666666666 | 82.35714285714286 | 84.83333333333333 |
| 6 | 137.16666666666666 | 138.875 | 83.3 | 80.625 |
| | None | None | None | None |
| 12 | 135.60714285714286 | 144.6 | 83.46428571428571 | 89.375 |
| | None | None | None | None |
| | None | None | None | None |
| | None | None | None | None |
| | None | None | None | None |
| 24 | 129.28571428571428 | 140.3 | 74.06666666666666 | 78.42857142857143 |
### Chart
| Category | | | | |
|---|---|---|---|---|
| 0 | 221.88095238095238 | 225.42857142857142 | 142.5952380952381 | 141.14285714285714 |
| 1 | 139.9189189189189 | 152.0 | 84.10810810810811 | 87.83333333333333 |
| 3 | 136.44117647058823 | 145.83333333333334 | 82.8529411764706 | 84.5 |
| 6 | 137.8709677419355 | 136.0 | 82.58064516129032 | 83.42857142857143 |
| | None | None | None | None |
| 12 | 139.93103448275863 | 141.28571428571428 | 84.6896551724138 | 85.14285714285714 |
| | None | None | None | None |
| | None | None | None | None |
| | None | None | None | None |
| | None | None | None | None |
| 24 | 136.375 | 124.8 | 76.23529411764706 | 72.8 | (mmHg)
 (mmHg)
Months
Months
C) RASi + CCB
### Chart
| Category | RASi(+)CCB(+) | RASi(+)CCB(-) | RASi(-)CCB(+) |
|---|---|---|---|
| 0 | 143.1290322580645 | 146.33333333333334 | 141.0909090909091 |
| 1 | 83.96296296296296 | 90.6 | 84.5 |
| 3 | 81.95652173913044 | 84.2 | 84.72727272727273 |
| 6 | 83.08333333333333 | 84.16666666666667 | 80.85714285714286 |
| | None | None | None |
| 12 | 82.63636363636364 | 86.5 | 91.14285714285714 |
| | None | None | None |
| | None | None | None |
| | None | None | None |
| | None | None | None |
| 24 | 74.36363636363636 | 73.25 | 79.66666666666667 |
### Chart
| Category | RASi(+)CCB(+) | RASi(+)CCB(-) | RASi(-)CCB(+) |
|---|---|---|---|
| 0 | 220.19354838709677 | 217.33333333333334 | 226.63636363636363 |
| 1 | 140.07407407407408 | 147.8 | 139.5 |
| 3 | 134.17391304347825 | 137.2 | 141.1818181818182 |
| 6 | 138.33333333333334 | 132.5 | 136.28571428571428 |
| | None | None | None |
| 12 | 134.36363636363637 | 140.16666666666666 | 157.42857142857142 |
| | None | None | None |
| | None | None | None |
| | None | None | None |
| | None | None | None |
| 24 | 134.2 | 117.0 | 140.0 | (mmHg)
Months
