# Supplementary material for: Impact of early initiation of renin-angiotensin blockade on renal function and clinical outcomes in patients with hypertensive emergency: a retrospective cohort study
Source: BMC Nephrol. 2023 Mar 22;24:68. doi: 10.1186/s12882-023-03117-1 (PMC10035153; doi:10.1186/s12882-023-03117-1)
Supplement: Supplementary file 3 — Additional file 3: Supplementary Fig. S2. Renal survival probabilities during 24-month follow-up. Urine-P/Cr; urinary protein/creatinine ratio, Ald; aldosterone, SBP; systolic blood pressure. [file 12882_2023_3117_MOESM3_ESM.pptx]

## Slide 1
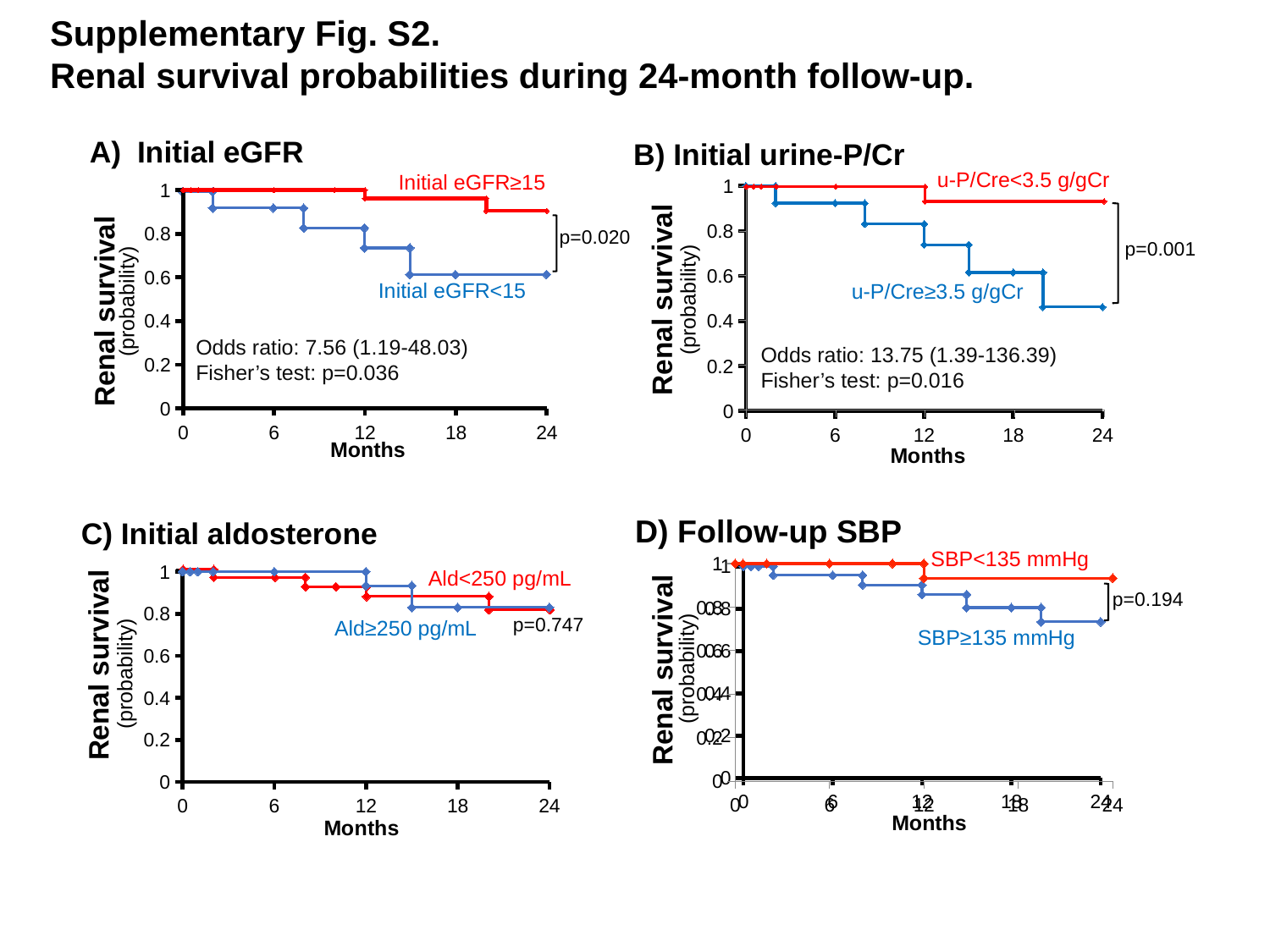

Supplementary Fig. S2.
Renal survival probabilities during 24-month follow-up.
### Chart
| Category | 生存率 |
|---|---|
### Chart
| Category | 生存率 |
|---|---|B) Initial urine-P/Cr
u-P/Cre<3.5 g/gCr
p=0.001
(probability)
u-P/Cre≥3.5 g/gCr
Odds ratio: 13.75 (1.39-136.39) Fisher’s test: p=0.016
Initial eGFR
### Chart
| Category | 生存率 |
|---|---|
### Chart
| Category | 生存率 |
|---|---|Initial eGFR≥15
p=0.020
Initial eGFR<15
(probability)
Renal survival
Odds ratio: 7.56 (1.19-48.03) Fisher’s test: p=0.036
Months
Renal survival
C) Initial aldosterone
### Chart
| Category | 生存率 |
|---|---|
### Chart
| Category | 生存率 |
|---|---|Ald<250 pg/mL
p=0.747
Ald≥250 pg/mL
(probability)
D) Follow-up SBP
### Chart
| Category | 生存率 |
|---|---|SBP<135 mmHg
### Chart
| Category | 生存率 |
|---|---|p=0.194
SBP≥135 mmHg
(probability)
Renal survival
Months
Renal survival
